# Physical Status and Parent-Child Feeding Behaviours in Children and Adolescents with Down Syndrome in The United Arab Emirates

**DOI:** 10.3390/ijerph16132264

**Published:** 2019-06-26

**Authors:** Tareq M. Osaili, Amita Attlee, Hira Naveed, Huda Maklai, Menna Mahmoud, Noor Hamadeh, Tooba Asif, Hayder Hasan, Reyad S. Obaid

**Affiliations:** 1 Department of Clinical Nutrition and Dietetics, College of Health Sciences, University of Sharjah, P.O. BOX 27272, Sharjah, UAE; 2 Department of Nutrition and Food Technology, Faculty of Agriculture, Jordan University of Science and Technology, P.O. BOX 27272, Irbid, Jordan; 3 Research Institute of Medical and Health Sciences, University of Sharjah, P.O. BOX 15551, Sharjah, UAE; 4 Nutrition and Health Department, College of Food and Agriculture, United Arab Emirates University, P.O. BOX 27272, Al Ain, UAE

**Keywords:** BMI, child feeding questionnaire, Down syndrome, feeding problem, parent-child feeding behaviour, physical status

## Abstract

The prevalence of Down syndrome (DS) in the United Arab Emirates (UAE) is high in comparison to the global statistics. The aim of this study is to assess the physical status, feeding problems, parent-child feeding relationship and weight outcome in children and adolescents with DS in the UAE. In this cross-sectional study, 83 individuals aged between 2–19 years with DS were recruited from three humanitarian centres for differently abled in the Emirates of Sharjah and Dubai, UAE. Socio-demographic characteristics; height, weight, BMI and body composition; feeding problems (STEP-CHILD screening tool); and parent-child feeding relationship (child feeding questionnaire—CFQ) were assessed. Correlations and regression analyses were used to determine the relationships and the best predictor of weight outcome (BMI) in DS participants. The median age of the participants was 9 (8) years. Fifty-five (66.3%) males and twenty-eight (33.7%) females constituted a sex ratio of 1.96:1. Five (6.2%) participants were short for their age, and 20.6% were overweight/obese compared to the growth charts for DS population. Body composition of females showed significantly higher percent body fat than males (25.5 (14.3)% vs. 18.2 (4.0)%, *p* = 0.03; 29.9 (2.8)% vs. 16.3 (12.2)%, *p* = 0.006) in 5–8.99 years and 12–19.99 years, respectively. The most common feeding difficulties on STEP-CHILD tool were food selectivity (62.2%), continued eating in the presence of food (57.7%) and swallowing without sufficient chewing (50%). Median score of total-CFQ for the parent-child feeding behaviour was 3.2 (1.9); parental restriction 3.3 (1.0); pressure to eat 3.0 (0.8); concern about child weight 3.7 (2.3). Parent-child feeding relationship was significantly positively correlated with feeding problems, and body weight of the participants. The best predictor for BMI was the parental concern about child weight (OR: 1.4, *p* = 0.02). The findings can be valuable for the health care professionals, parents and caretakers of children and adolescents with DS in emphasizing the need for regular monitoring of their physical status, and feeding behaviours. In addition, it reinforces the role of parents in mindfully managing their child feeding relationship in promoting healthy eating behaviours and weight of their youth with DS.

## 1. Introduction

Down syndrome (DS), resulting from the trisomy of chromosome 21, is the most common chromosomal disorder, with a global prevalence ranging from 6.1 to 13.1 per 10,000 people in the USA [1]. In the United Arab Emirates (UAE), DS is reported to occur in 1 out of 319 (31.3 in 10,000 live births) Emiratis and 1 out of 602 (16.6 out of 10,000) non-Emiratis [2]. According to the National Down Syndrome Society of America [3], DS occurs in people of all races and economic levels.

DS is characterized by several physiological and biochemical changes, cognitive disability and growth retardation, thyroid dysfunction, low muscle tone, cardiac complications, and feeding problems [4]. Short stature is a cardinal feature [5], and lower weight for stature ratios indicate physical growth retardation in children with DS [6]. Even though, puberty appears somewhat early, DS individuals have a decreased pubertal growth rate on the charts [5]. Besides, the development of gross and fine motor skills in individuals with DS is slow and continually lags behind that of their typically developing counterparts [7]. This fact coupled with inappropriate parental feeding practices can result in feeding difficulties and delayed development of feeding skills in the children with DS [8]. 

The prevalence rates of feeding problems were much higher in children with special needs (nearly one-thirds of the total samples) as compared to those developing typically [9]. These feeding problems varied from food selectivity or texture to more severe problems like food refusal and dysphagia [10]. 

Shorter stature and high rates of overweight and obesity in the DS population emphasize the need of healthy eating habits and balanced diets in children with DS [11,12]. Parents can have both an encouraging and restrictive impact on the type and quantity of foods their children consume [13]. For instance, “overly permissive” actions by parents such as infrequent insistence on eating during meals, or frequent preparation of special meals for children different than the family meals explained over 34% of the links between children’s feeding problems and weight and diet related outcomes [9]. 

Despite a high prevalence of DS in the UAE and the Gulf region, evidence on feeding behaviours of individuals with DS and parent-child feeding relationship is mostly limited to Western studies. Thus, the present study was undertaken to determine the physical status, feeding problems, parent-child feeding relationship and weight outcome in children and adolescents with DS in the UAE.

## 2. Methods

### 2.1. Design and Participants

A cross-sectional research design was used. The study was carried out in three main humanitarian centres for differently abled individuals namely, Sharjah City for Humanitarian Services in Sharjah, and Rashid Centre for People of Determination and Al Noor Training Centre for Persons with Disabilities in Dubai, UAE. A total of 83 enrollees aged 2–19 years with DS participated in the present study alongside with parents. The ethical approval on the research protocol and consent form was obtained from the Research Ethics Committee, University of Sharjah, UAE (REC-18-02-28-01-S). Administrative approvals were taken from each institute to access the participants. Written informed consent was obtained from the parents of the participants prior to data collection.

### 2.2. Data Collection

The data was collected between February 2018–April 2018. Information about the participants with DS was obtained from their parents. 

#### 2.2.1. Survey: Socio-Demographic

Socio-demographic characteristics and health conditions: Participant’s age, sex, nationality, presence of health conditions (such as celiac disease, lactose intolerance, gastro-esophageal reflux, allergies, constipation) were asked. 

#### 2.2.2. Anthropometric Measurements

Height: A portable lightweight stadiometer (Seca 213, Hamburg, Germany) with a fixed vertical backboard and an adjustable headpiece against a wall was used to measure the standing height to the nearest 0.1 cm. The participants were assisted to stand erect with both feet flat on the platform, the back of the head, shoulder blades, buttocks, and heels making contact with the backboard. In case of difficulty in standing erect and following the instructions, assistance was sought from the centre’s nurse. The measurements were recorded after the participants’ heads were aligned in the Frankfort horizontal plane. The mean of three consecutive readings was considered [14]. 

Body Composition: Body Composition Analyzer (BCA) (Tanita BC-420MA, Tokyo, Japan) was used to measure the body composition (weight, BMI, fat mass, percent body fat—%BF, fat free mass, muscle mass, total body water, and percent total body water—%TBW) using electrical impedance [15]. Participants below 5 years of age were excluded as per the specification of this equipment that includes FDA cleared body fat analysis for children aged 5 years and above. 

The physical status of participants was assessed from the above anthropometric measurements using appropriate nutritional indices—height for age, and BMI for age. CDC (2000) growth charts [16] as well as the charts specific to DS population [17] were used to compare the physical growth of the participants. The following criteria used for typically developing children and adolescents (<20 years of age) was adopted to categorize the nutritional status of the participants [16]: 

Stature (Height) for age percentile cut-offs: Short stature < 5th, Normal 5–94th, Tall ≥ 95th

BMI-for-age percentile cut-offs: Underweight < 5th, Healthy weight 5–84th, Overweight 85–94th, Obese ≥95th.

#### 2.2.3. Survey: Feeding Problems

A 15-item questionnaire, namely “Screening Tool of Feeding Problems”, applied to children (STEP-CHILD) was used [9] to assess the frequency of occurrence of the feeding problems. Frequency was rated on a 3-point Likert-type scale wherein a rating of “0” = no occurrence of the behaviour, “1” = the behaviour occurred between 1 and 10 times, and “2” = the behaviour has occurred more than 10 times per month [18]. STEP-CHILD has six subscales of child feeding problems: chewing difficulties, rapid eating, food refusal, food selectivity, vomiting, and stealing food. The six subscales showed a mean internal reliability of Cronbach’s alpha 0.62 [9]. Total feeding problems in the participants were determined by the sum of responses to 15-STEP-CHILD items with scores ranging from 0–30.

#### 2.2.4. Survey: Parent-Child Feeding Relationship

Child Feeding Questionnaire (CFQ) is considered an acceptable measure to assess child-feeding perceptions, attitudes, and child weight status [19]. CFQ comprises of seven subscales with a mean internal consistency of Cronbach’s alpha 0.79 [19]. In the current study, only 3 subscales of the seven subscales from the CFQ were used to measure parent-child feeding relationship. These subscales were chosen based on applicability to the study aims, previously documented validity, internal consistency and to decrease the burden on the study participants [20]. 

#### 2.2.5. Parental Feeding Behaviours

Two subscales were used to measure parental feeding behaviours. The first subscale is ‘Restriction’ which measures the parents’ restriction of the child’s access to foods including type and amount of foods (eight items). The second subscale is ‘Pressure to eat’ which measures pressuring the child to eat by encouraging increased amount and type of foods (four items). A 5-point Likert-type scale was used for these subscales ranging from “never” to “always”, coinciding with 1–5 respectively. Further, the third subscale, ‘Concern about body weight’ (three items) was used to measure parental concerns about their child’s weight. This subscale measures the parent’s level of concern about the child being overweight. A 5-point Likert-type scale was used for the third subscale ranging from “extremely unconcerned” to “extremely concerned”, coinciding with 1 to 5, respectively. 

The scores of items in each subscale were added together and divided by the number of questions in each respective subscale. The scores were also added for the used CFQ subscales and divided by the total number of items within CFQ. Medians and inter-quartile ranges (IQR) were then calculated using these values for each subscale as well as the total CFQ scores [21]. 

### 2.3. Statistical Analysis

Data was analysed by using Statistical Package for the Social Sciences (SPSS Version 21, Chicago, IL, USA). The data was checked for normality using the Shapiro-Wilk test. Non-parametric statistical tests were applied. Data were presented as medians and interquartile ranges (IQR), and percentages. Mann-Whitney U test was used to compare the child feeding problems and parent-child feeding relationship scores between the sexes, and Chi-Square test to find the association between frequency distributions and age groups. Spearman’s rank correlation coefficient (*r*) was performed to assess the relationships in the variables of physical status, parental feeding behaviour and child feeding problems. Multivariate binary logistic regression was performed for the prediction of BMI in participants. A *p* value < 0.05 was considered as statistically significant. 

## 3. Results

### 3.1. Socio-Demographic Considerations and Health Conditions

Table 1 shows the distribution of 83 participants according to age and sex from the three centres in the Emirates of Sharjah and Dubai, UAE. The participants ranged from 2 years to 19 years with the median and IQR age of 9 (8) years. Fifty-five (66.3%) males and 28 (33.7%) females constituted a sex ratio of 1.96:1 in this study. 

Nearly one-fifth (n = 16) of the participants were UAE Nationals (Emiratis); the rest were expatriate Arabs from middle-east countries (40; 48.2%) and other nationalities from South-East Asia (27; 32.5%). 

Parents reported health conditions of allergy (9.6%), gastro-oesophageal reflux (7.2%), thyroid dysfunction (2.4%), hearing deficit (2.4%), lactose intolerance (1.2%), and asthma (1.2%) in the participants. Fifty percent of the total allergy cases were reported to be due to food. Further, constipation, per se, was reported to be a common health condition in almost 39.5% of the participants with frequencies between “sometimes” and “often”. 

### 3.2. Physical Status

Median and IQR values of weight and height in the age categories 2–4.99 years, 5–8.99 years, 9–11.99 years and 12–19.99 years were 14.7 (5.8) kg and 94.0 (11.7) cm; 21.8 (9.4) kg and 111.5 (19.7) cm; 33.6 (14.2) kg and 126.4 (20.6) cm; 56.4 (21.3) kg and 146.5 (12.0) cm, respectively. 

Regarding height for age of the participants, the majority (61.2%) fell short in stature for age when compared with the reference growth charts for typically developing children. On the contrary, most of the participants (87.5%) attained normal heights in comparison with the special growth charts developed for children with DS. Only 6.2% participants were short for their age. Similar trend was observed in terms of BMIs of 78 participants. The median and IQR values were 16.0(2.6) kg/m^2^ in 2–4.99 years, 17.5(4.9) kg/m^2^ in 5–8.99 years, 21.4(5.8) kg/m^2^ in 9–11.99 years and 26.3(6.3) kg/m^2^ in 12–19.99 years. When compared with the reference growth charts of BMI for age for typically developing children, the majority of participants (57.7%) were found to be either overweight or obese. However, a more realistic picture was revealed when the growth charts for children with DS were used for comparison, indicating that 71.7% children were maintaining their body weights in the typical range for DS. Yet, 20.6% of the participants were categorized as either overweight or obese (Table 2).

Table 3 presents the body composition of the participants aged 5–19.99 years. Significant sex differences were noted in the %BF and %TBW at 5.0–8.99 years and 12.0–19.99 years. While females had significantly higher %BF (25.5 (14.3)% vs. 18.2 (4.0)%, *p* = 0.03; 29.9 (2.8)% vs. 16.3 (12.2)%, *p* = 0.006), %TBW was found to be significantly higher in male than the female participants (60.0 (2.9)% vs. 54.8 (10.8) *p* = 0.03; 61.8 (8.8)% vs. 51.3 (3.9)%, *p* = 0.005).

### 3.3. Feeding Problems (STEP-CHILD)

Table 4 summarizes the frequency of feeding problems in the participants on the 15-item STEP-CHILD screening tool. Feeding problems were reported by the parents of 82/83 participants. The most frequently documented feeding difficulties were related to food selectivity (62.2%), continued eating in the presence of food (57.7%) and swallowing with insufficient chewing of food (50.0%). Significant sex differences were found in two specific items, 42.6% males in contrast to 39.3% females ate large meals in short time, and 44.4% males versus 17.9% females ate certain textures of food only (*p* = 0.02). Significant correlations were found between feeding problems and age of the participants in terms of inability to eat independently (*p* = 0.004), and pushing away food or attempting to leave the area (*p* = 0.001). Frequencies of these problems showed a decreasing trend from toddler stage through adolescence, that is, 84.6%, 44.4%, 26.7%, 18.5% for dependence in eating and 53.8%, 25.9%, 14.7%, 13.4% for pushing/leaving food, respectively.

The median, IQR value of total feeding problems was 13.0(7.0). Among the six subscales, the highest score was reported in rapid eating (4.0(3.0)) followed by difficulty in chewing (3.0(4.0)), food refusal (2.0(3.0)) and food selectivity (2.0(2.0)) skills. No significant differences existed between the sexes (Table 5) and different age groups (Table 6).

### 3.4. Parent-Child Feeding Relationship (CFQ)

Table 7 and Table 8 summarize the results of CFQ on the three selected subscales. The three most frequently (often and always) reported items by the parents in the “restriction” subscale were “If I did not guide or regulate my child’s eating, she/he would eat too many of her/his favourite foods” (56.0%), “If I did not guide or regulate my child’s eating, she/he would eat too many junk foods. (chips, pizza, burger, French fries, sweets)” (53.7%) and “I intentionally keep some food out of my child’s reach” (52.5%). The parental behaviour of “pressure to eat” towards the participants was also evident from the most frequently reported items “I have to be especially careful to make sure my child eats enough” (68.3%) and “My child should always eat all of the food on his/her plate” (53.6%). Further, the “concern about child weight” was obvious; 67.1% parents affirmed when asked “How concerned are you about your child becoming over weight?” Parent-child feeding relationship was independent of sex and age categories. 

The median score of total parental control on CFQ was 3.2(1.9) and the averages for each subscale were: restriction 3.3(1.0); pressure to eat 3.0(0.8); concern about child weight 3.7(2.3). There were no significant differences between M and F on the 3 subscales (Table 9).

### 3.5. Correlations of Physical Status, Feeding Problems and Parent-Child Feeding Relationship

Spearman’s rank correlation coefficients tested on 78 participants were significant for various parameters (Table 10). Parental control was found to have a direct influence on the feeding problems of the participants. To elaborate, parental feeding behaviour measured through the total CFQ score was directly related to the problem of rapid eating (r = 0.32, *p* < 0.001) in the participants. Specifically, the restrictive behaviour of the parents directly influenced rapid eating (r = 0.41, *p* < 0.001), and parental pressure to eat correlated positively with the chewing problems (r = 0.25, *p* < 0.05) in their children and adolescents. On the other hand, there was an inverse correlation between the parental concern about child weight and the chewing problems (r = −0.27, *p* < 0.001) in the participants. 

Moreover, parental feeding behaviour in terms of total-CFQ score, and concern about child weight subscale were positively related to the BMI (r = 0.31 and r = 0.41 respectively; *p* < 0.001) of the participants. However, BMI was inversely related (r = −0.29; *p* < 0.001) to chewing problems in the participants. 

### 3.6. Weight Outcome

Table 11 summarizes the binary logistic regression and highlights that the best predictor of weight outcome (BMI) in the participants was the parental concern about child’s body weight (OR: 1.40, *p* = 0.02).

## 4. Discussion

The sex ratio of 1.96:1 favouring males was supported by a previous study in the UAE with the male-to-female ratio of 1.6:1 [2] and 1.69 with 63% male representation in a clinic based study in the UAE [3]. Earlier Verma and Huq [22] also reported the ratio of 1.3:1. Data from 55 publications [23]. found that the sex ratio was generally skewed towards males in the majority of studied populations. 

DS individuals have a high frequency of infections, characterized by increased severity and prolonged course of disease, partially attributed to defects of the immune system and non-immunological factors such as abnormal anatomical structures [24]. Nearly one-tenth of the participants were reported to have allergies in the present study. Development of different mechanisms of allergies in DS individuals has been suggested as compared to the general population [25]. Thyroid dysfunction was reported by the parents in 4.3% of the participants. Anderson et al. also reported that children with DS are at high risk for autoimmune phenomenon such as hypothyroidism [26]. Constipation was reported with the frequencies of “often” and “always” occurrence in almost 40% of the participants. Hickey et al. [27] suggested that constipation is a widespread functional gastrointestinal symptom occurring in 30% to 50% of DS population.

Similar to the typically developing children and adolescents, it is important to assess and monitor the physical status of those with DS. To reiterate, the physical growth of the participants in the present study were compared with the growth charts for typically developing children [16] as well as the specific growth charts developed for 2–20 years old with DS [17]. Lower height and higher BMI in youth aged 2–20 years with DS in comparison to CDC growth charts necessitated the need of specific growth charts for DS youth [17]. As expected, the participants fared better and “normal” when compared with the specific charts for DS (87.5%) in contrast to 37.5% only with CDC reference for height for age. Similarly, 71.7% were categorized with healthy weight (BMI for age) according to the growth charts for DS population in contrast to 37.2% as per the CDC growth charts. The recent growth charts published for children from birth up to 20 years with DS in Brazil also presented lower growth levels below those described at all developmental phases for typically developing counterparts; this divergence in the average of height-for-age was pronounced at 12–15 years and continued to increase, reaching its greatest level at 17–19 years [28]. 

There is a tendency to deviate from the normal body weight in children with DS. According to Grammatikopoulou et al. [29], children with DS are genetically predisposed to becoming overweight and obese. This fact was reiterated in a study conducted in US suggesting that obesity is a significant problem in children with DS and that it is more prevalent than in the typically developing children [30]. A previous study also showed that individuals with DS were not only shorter in stature, they also had a higher BMI with more than half of them identified as obese than their siblings [7]. The present study reinforced weight problems of overweight and obesity in 10.3% each, and underweight in 7.7% participants when compared with the DS growth charts. Results from Rodrigues et al. [31] also found that 22% of DS participants were overweight while 30% had low weight. Earlier, Fernhall et al. [32] showed that 33.3% of the adolescents with DS were overweight, 33.3% were malnourished and 33.3% were well-nourished. Further, the children with DS were significantly shorter and heavier than the typical children in the UAE [2].

The children and adolescents with DS have a unique body composition, and higher %BF than their counterparts without DS [33]. The findings of their study in Spain on adolescents aged 12–18 years showed %BF in males (18.8 (4.9)%) and females (29.1 (4.7)%) similar to those of the present UAE study (males 16.3 (12.2)%; females 29.9 (2.8)%). 

The origin of obesity in the population with DS should not be entirely attributed to the syndrome itself [7]. The potential causes of obesity may include slower basal metabolic rate, the compulsive feeding due to difficulty in chewing, and the general hypotony of the muscles causing less satiation after meals resulting in overeating [31]. Previous study [34] estimated that up to 80% of the children with DS compared to 25% typical population have difficulties related to food or feeding. The feeding problem related to food selectivity was reported by 62.2% of the parents in the present study. Rezaei et al. [18] found that 73.6% of the participants depicted food selectivity, and 79.2% showed feeding skills disorders. Besides, swallowing without sufficient chewing was reported in 50.0% of the participants in the present study. Moreover, chewing problems were significantly correlated with age, the problem decreasing with increasing age (r = −0.33; *p* < 0.001). Our findings were similar to the children with special needs (r = −0.22; *p* < 0.008) in the US [9]. In addition, the present study found significant negative correlations between chewing problems and weight, height (shortness), BMI and parental concern about child weight; and positive relationships with other feeding problems including food refusal and selectivity. These problems may be attributed to the parental child feeding behaviour as reflected by the significant correlations of chewing problems in the participants with the restriction and pressure to eat imposed by the parents. 

Further, 57.3% of the parents in the present study reported that their child, often or always, continued to eat as long as food was presented without self-limiting, implying that overeating may be common among DS population, a potential factor contributing to weight gain. 

The fact that the parents are aware about the problems of overweight and obesity in children with special needs was also recognized in the present study on DS participants. As mentioned, 67.1% of the parents claimed to be either very concerned or fairly concerned about their child’s body weight. This parental concern about child weight was significantly related to the restrictive behaviour in the parents. Evidence in the literature has demonstrated how controlling feeding behaviours could be counterproductive and can lead to weight gain [35,36]. O’Neill et al. [37] reported that imposing continuous control in feeding may be a greater risk for weight gain as children learn to ignore hunger and satiety cues. 

Recently, Schmidt et al. [38] presented the CFQ subscales’ scores indicating the parental control as a function of child weight categories in a typical population based cohort in Germany. The average scores of restriction [3.3 (1.0)] and concern about child weight [3.7 (2.3)] subscales in the present study corresponded with the restriction [3.43 (1.04)] and concern [(3.38 (1.19)] imposed on the German children in overweight category. Similarly, pressure to eat [3.0 (0.8)] subscale score in the present study was much beyond the score of 1.44 (0.66) reported for the obese category in German children. Though the comparison here is made with the CFQ scores of typically developing children, the above subscale scores in the present study imply that the parent-child feeding behaviour may be attributed to weight gain in children with DS as well [38].

The present study highlighted that the best predictor of weight outcome (BMI) in the participants was parental concern of child weight. However, Berkowitz et al. (2010) found rapid eating as the strong predictor of weight gain [39].

## 5. Conclusions

Malnutrition in terms of shortness for age and deviation from normal body weight in the forms of overweight and obesity are common in children and adolescents with DS in the UAE. Feeding problems in them are prominent and associated with counterproductive parental-child feeding behaviours that may be restrictive, induce pressure to eat, and/or increase concern about child weight. The findings of our study can be valuable in the future interventions for the health care professionals, parents and caretakers of the children and adolescents with DS in emphasizing the need for regular monitoring of their physical status and feeding behaviours. In addition, it reinforces the role of parents in mindfully managing their child feeding relationship in promoting healthy eating behaviours and body weights of their youth with DS. 

## Figures and Tables

**Table 1:** Distribution of participants according to age and sex (n = 83).

Category	Age Group	Male (n = 55) % (n)	Female (n = 28) % (n)
Toddlers & pre-school age	2–4.99 years	12.7 (7)	25.0 (7)
Middle childhood	5–8.99 years	29.1 (16)	39.3 (11)
Preadolescence	9–11.99 years	21.8 (12)	10.7 (3)
Adolescence	12–19.99 years	36.4 (20)	25.0 (7)

**Table 2:** Distribution of participants according to height-for-age (n = 80) and BMI-for-age percentile growth charts (n = 78).

Chart Reference	Height for Age (n = 80)			BMI for Age (n = 78)			
	Short	Normal	Tall	Underweight	Normal	Overweight	Obese
CDC % (n)	61.2 (49)	37.5 (30)	1.3 (1)	5.1 (4)	37.2 (29)	32.1 (25)	25.6 (20)
DS % (n)	6.2 (5)	87.5 (70)	6.3 (5)	7.7 (6)	71.7 (56)	10.3 (8)	10.3 (8)

**Table 3:** Medians and interquartile ranges (IQR) of body composition in participants aged 5–19.99 years.

Age Groups (Years)	5.00–8.99 Median (IQR)			9.00–11.99 Median (IQR)			12.00–19.99 Median (IQR)		
	M (n = 11)	F (n = 9)	*p* Value	M (n = 9)	F (n = 2)	*p* Value	M (n = 16)	F (n = 6)	*p* Value
Fat mass (kg)	3.7 (2.6)	4.9 (5.6)	0.22	8.3 (8.0)	7.7 (-)	1.00	9.5 (9.3)	16.2 (6.1)	0.03
Percent Body Fat (%)	18.2 (4.0)	25.5 (14.3)	0.03	23.1 (20.5)	22.4 (-)	0.81	16.3 (12.2	29.9 (2.8)	0.006
Fat free mass (kg)	18.0 (6.2)	17.4 (6.80)	0.85	27.7 (8.0)	26.0 (-)	0.63	44.6 (18.5)	38.8 (10.9)	0.46
Muscle mass (kg)	16.9 (5.9)	16.5 (6.45)	0.85	26.2 (7.6)	28.2 (-)	0.63	42.3 (17.7)	36.9 (7.3)	0.34
Total body water (kg)	13.2 (4.5)	12.7 (5.0)	0.85	20.3 (5.9)	19.0 (-)	0.55	32.6 (13.9)	28.5 (4.4)	0.30
Percent Total body water (%)	60.0 (2.9)	54.8 (10.8)	0.03	56.3 (15.1)	55.2 (-)	0.63	61.8 (8.8)	51.3 (3.9)	0.005

**Table 4:** Distribution of participants according to frequency of reported feeding problems on STEP-Child screening tool (n = 82).

Variable	Not at All % (n)	1–10 Times/Month % (n)	>10 Times/Month % (n)
My child cannot independently feed	40.2 (33)	20.7 (17)	39.0 (32)
My child does not demonstrate ability to chew.	52.4 (43)	8.5 (7)	39.0 (32)
My child swallows without chewing sufficiently.	29.3 (24)	20.7 (17)	50.0 (41)
My child only eats a small amount of food presented.	28.0 (23)	30.5 (25)	41.5 (34)
My child will continue to eat as long as food is presented	28.0 (23)	14.6 (12)	57.3 (47)
My child eats large amounts in short time.	35.4 (29)	23.2 (19)	41.5 (34)
My child’s problem behaviours increase during meals.	50.0 (41)	25.6 (21)	24.4 (20)
My child pushes food away or attempts to leave area.	50.0 (41)	25.6 (21)	24.4 (20)
My child only eats foods at certain temperature.	36.6 (30)	24.4 (20)	39.0 (32)
My child will only eat select types of foods.	17.1 (14)	20.7 (17)	62.2 (51)
My child only eats certain textures.	37.8 (31)	26.8 (22)	35.4 (29)
My child regurgitates or re-swallows food.	76.8 (63)	13.4 (11)	9.8 (8)
My child vomits during or right after meals.	82.9 (68)	9.8 (8)	7.3 (6)
My child steals or attempts to steal food during meals.	73.2 (60)	11.0 (9)	15.9 (13)
My child steals or attempts to steal food outside mealtimes.	67.1 (55)	11.0 (9)	22.0 (18)

**Table 5:** Medians and interquartile ranges (IQR) of total STEP-Child and six subscale scores.

Subscale Category	Total Sample (n = 82) Median (IQR)	Males (n = 54) Median (IQR)	Females (n = 28) Median (IQR)	*p* Value
Chewing (3 items)	3.0(4.0)	4.0(4.0)	2.5(4.0)	0.60
Rapid Eating (3 items)	4.0(3.0)	3.5(2.0)	4.0(2.75)	0.78
Food Refusal (3 items)	2.0(3.0)	1.0(3.75)	2.0(3.0)	0.79
Food Selectivity (2 items)	2.0(2.0)	2.5(4.0)	2.0(1.0)	0.41
Vomiting (2 items)	0.0(0.25)	0.0(1.0)	0.0(0.0)	0.26
Stealing Food (2 items)	0.0(2.0)	0.0(1.75)	0.0(2.0)	0.44
Total	13.0(7.0)	12.5(10)	13.5(7.5)	0.72

**Table 6:** Medians and interquartile ranges (IQR) of total STEP-Child and age subscale scores.

	Age Groups in Years				
Subscale Category	2.00–4.99 n = 13 Median (IQR)	5.00–8.99 n = 27 Median (IQR)	9.00–11.99 n = 15 Median (IQR)	12.00–19.99 n = 27 Median (IQR)	*p* Value
Chewing (3 items)	4.0(5.0)	4.0(5.0)	3.0(4.0)	2.0(3.0)	0.25
Rapid Eating (3 items)	4.0(3.5)	3.0(2.0)	5.0(2.0)	4.0(2.0)	0.17
Food Refusal (3 items)	4.0(2.5)	2.0(2.0)	3.0(4.0)	2.0(2.0)	0.07
Food Selectivity (2 items)	2.0(2.0)	3.0(3.0)	2.0(2.0)	2.0(3.0)	0.41
Vomiting (2 items)	0(1.0)	0(2.0)	0(1.0)	0(0)	1.00
Stealing Food (2 items)	0(1.25)	0(2.0)	0(2.0)	0(2.0)	0.43
Total	3.0(4)	2.5(3.25)	2.5(3.5)	2.0(2.5)	0.46

**Table 7:** Distribution of overall participants according to frequency of reported parent-child feeding relationship on Child Frequency Questionnaire (n = 82).

**Variable**	**Never** **% (n)**	**Rarely** **% (n)**	**Sometimes % (n)**	**Often % (n)**	**Always** **% (n)**
**Restriction**					
I have to be sure that my child does not eat too many sweets. (candy, ice cream, cake, pastries)	7.3(6)	12.2(10)	41.5(34)	19.5(16)	19.5 (16)
I have to be sure that my child does not eat too many fatty foods. (cheese, butter, pizza, burger)	14.6(12)	14.6(12)	30.5(25)	22.0(18)	18.3 (15)
I have to be sure that my child does not eat too much of his/her favourite foods.	13.4(11)	12.2(10)	30.5(25)	23.2(19)	20.7 (17)
I intentionally keep some food out of my child’s reach.	20.7(17)	6.1 (5)	20.7(17)	29.3(24)	23.2 (19)
I offer sweets (candy, ice cream, cake, pastries) to my child as a reward for good behaviour.	23.2(19)	14.6(12)	31.7(26)	18.3(15)	12.2 (10)
I offer my child his/her favourite food in exchange for good behaviour.	20.7(17)	18.3(15)	31.7(26)	18.3(15)	11.0 (9)
If I did not guide or regulate my child’s eating, she/he would eat too many junk foods. (chips, pizza, burger, French fries, sweets).	17.1(14)	13.4(11)	15.9(13)	30.5(25)	23.2 (19)
If I did not guide or regulate my child’s eating, she/he would eat too many of her/his favourite foods.	12.2(10)	8.5 (7)	23.2(19)	28.0(23)	28.0 (23)
**Pressure to eat**					
My child should always eat all of the food on his/her plate.	12.2(10)	11.0 (9)	23.2(19)	28.0(23)	25.6 (21)
I have to be especially careful to make sure my child eats enough	7.3 (6)	11.0 (9)	13.4(11)	30.5(25)	37.8 (31)
If my child says I’m not hungry, I try to get him/her to eat anyway.	56.1 (46)	15.9(13)	17.1(14)	6.1 (5)	4.9 (4)
If I did not guide my child’s eating, she/he would eat much less than she/he should.	32.9(27)	15.9(13)	26.8(22)	12.2(10)	12.2 (10)
	Extremely unconcerned % (n)	Unconcerned % (n)	Slightly concerned % (n)	Moderately concerned % (n)	Extremely concerned % (n)
**Concern about child weight**					
How concerned are you about your child eating too much when you are not around her/him?	23.2(19)	3.7 (3)	24.4(20)	17.1(14)	31.7 (26)
How concerned are you about your child having to diet to maintain a desirable weight?	24.4(20)	3.7 (3)	23.2(19)	22.0(18)	26.8 (22)
How concerned are you about your child becoming over weight?	13.4(11)	2.4 (2)	17.1(14)	18.3(15)	48.8 (40)

**Table 8:** Distribution of participants according to frequency of reported parent-child feeding relationship on Child Frequency Questionnaire (n = 82).

**Variable**	**2.00–4.99 Years**					**5.00–8.99 Years**				
	**Never**	**Rarely**	**Sometimes**	**Often**	**Always**	**Never**	**Rarely**	**Sometimes**	**Often**	**Always**
	**% (n)**	**% (n)**	**% (n)**	**% (n)**	**% (n)**	**% (n)**	**% (n)**	**% (n)**	**% (n)**	**% (n)**
**Restriction**										
I have to be sure that my child does not eat too many sweets. (candy, ice cream, cake, pastries)	15.4 (2)	15.4 (2)	23.1 (3)	30.8 (4)	15.4 (2)	3.8 (1)	15.4 (4)	50.0 (13)	11.5 (3)	19.2 (5)
I have to be sure that my child does not eat too many fatty foods. (cheese, butter, pizza, burger)	15.4 (2)	15.4 (2)	38.5 (5)	23.1 (3)	7.7 (1)	8.0 (2)	16.0 (4)	32.0 (8)	24.0 (6)	20.0 (5)
I have to be sure that my child does not eat too much of his/her favourite foods.	0 (0)	16.7 (2)	25.0 (3)	33.3 (4)	25.0 (3)	3.8 (1)	15.4 (4)	26.9 (7)	26.9 (7)	26.9 (7)
I intentionally keep some food out of my child’s reach.	7.7 (1)	7.7 (1)	30.8 (4)	38.5 (5)	15.4 (2)	25.0 (6)	4.2 (1)	20.8 (5)	25.0 (6)	25.0 (6)
I offer sweets (candy, ice cream, cake, pastries) to my child as a reward for good behaviour.	30.8 (4)	0 (0)	53.8 (7)	15.4 (2)	0 (0)	15.4 (4)	15.4 (4)	26.9 (7)	34.6 (9)	7.7 (2)
I offer my child his/her favourite food in exchange for good behaviour.	30.8 (4)	15.4 (2)	46.2 (6)	0 (0)	7.7 (1)	23.1 (6)	19.2 (5)	23.1 (6)	26.9 (7)	7.7 (2)
If I did not guide or regulate my child’s eating, she/he would eat too many junk foods. (chips, pizza, burger, French fries, sweets).	8.3 (1)	8.3 (1)	16.7 (2)	58.3 (7)	8.3 (1)	7.7 (2)	19.2 (5)	19.2 (5)	23.1 (6)	30.8 (8)
If I did not guide or regulate my child’s eating, she/he would eat too many of her/his favourite foods.	7.7 (1)	7.7 (1)	7.7 (1)	46.2 (6)	30.8 (4)	4.0 (1)	16.0 (4)	32.0 (8)	20.0 (5)	28.0 (7)
**Pressure to eat**										
My child should always eat all of the food on his/her plate.	16.7 (2)	8.3 (1)	66.7 (8)	8.3 (1)	0 (0)	15.4 (4)	19.2 (5)	19.2 (5)	30.8 (8)	15.4 (4)
I have to be especially careful to make sure my child eats enough	15.4 (2)	0 (0)	30.8 (4)	38.5 (5)	15.4 (2)	0 (0)	15.4 (4)	11.5 (3)	26.9 (7)	46.2 (12)
If my child says I’m not hungry, I try to get him/her to eat anyway.	30.8 (4)	23.1 (3)	23.1 (3)	7.7 (1)	15.4 (2)	57.7 (15)	11.5 (3)	19.2 (5)	7.7 (2)	3.8 (1)
If I did not guide my child’s eating, she/he would eat much less than she/he should.	15.4 (2)	7.7 (1)	38.5 (5)	15.4 (2)	23.1 (3)	30.8 (8)	7.7 (2)	38.5 (10)	11.5 (3)	11.5 (3)
**Concern about child weight**	**Extremely unconcerned**	**Unconcerned**	**Slightly concerned**	**Moderately concerned**	**Extremely concerned**	**Extremely unconcerned**	**Unconcerned**	**Slightly concerned**	**Moderately concerned**	**Extremely concerned**
How concerned are you about your child eating too much when you are not around her/him?	38.5 (5)	0 (0)	23.1 (3)	23.1 (3)	15.4 (2)	29.6 (8)	3.7 (1)	29.6 (8)	7.4 (2)	29.6 (8)
How concerned are you about your child having to diet to maintain a desirable weight?	23.1 (3)	0 (0)	30.8 (4)	38.5 (5)	7.7 (1)	25.9 (7)	7.4 (2)	22.2 (6)	18.5 (5)	25.9 (7)
How concerned are you about your child becoming over weight?	23.1 (3)	0 (0)	15.4 (2)	38.5 (5)	23.1 (3)	18.5 (5)	0 (0)	33.3 (9)	7.4 (2)	40.7 (11)
**Variable**	**9.00–11.99 years**						**12.00–19.99 years**			
	**Never**	**Rarely**	**Sometimes**	**Often**	**Always**	**Never**	**Rarely**	**Sometimes**	**Often**	**Always**
	**% (n)**	**% (n)**	**% (n)**	**% (n)**	**% (n)**	**% (n)**	**% (n)**	**% (n)**	**% (n)**	**% (n)**
**Restriction**										
I have to be sure that my child does not eat too many sweets. (candy, ice cream, cake, pastries)	6.7 (1)	0 (0)	20.0 (3)	33.3 (5)	40.0 (6)	3.7 (1)	14.8 (4)	55.6 (15)	14.8 (4)	11.1 (3)
I have to be sure that my child does not eat too many fatty foods. (cheese, butter, pizza, burger)	6.7 (1)	13.3 (2)	20.0 (3)	20.0 (3)	40.0 (6)	18.5 (5)	14.8 (4)	33.3 (9)	22.2 (6)	11.1 (3)
I have to be sure that my child does not eat too much of his/her favourite foods.	20.0 (3)	6.7 (1)	33.3 (5)	20.0 (3)	20.0 (3)	18.5 (5)	11.1 (3)	37.0 (10)	18.5 (5)	14.8 (4)
I intentionally keep some food out of my child’s reach.	20.0 (3)	0 (0)	6.7 (1)	40.0 (6)	33.3 (5)	14.8 (4)	11.1 (3)	25.9 (7)	25.9 (7)	22.2 (6)
I offer sweets (candy, ice cream, cake, pastries) to my child as a reward for good behaviour.	26.7 (4)	26.7 (4)	20.0 (3)	13.3 (2)	13.3 (2)	22.2 (6)	14.8 (4)	33.3 (9)	7.4 (2)	22.2 (6)
I offer my child his/her favourite food in exchange for good behaviour.	20.0 (3)	20.0 (3)	33.3 (5)	13.3 (2)	13.3 (2)	11.1 (3)	18.5 (5)	33.3 (9)	22.2 (6)	14.8 (4)
If I did not guide or regulate my child’s eating, she/he would eat too many junk foods. (chips, pizza, burger, French fries, sweets).	20.0 (3)	6.7 (1)	13.3 (2)	33.3 (5)	26.7 (4)	22.2 (6)	14.8 (4)	14.8 (4)	25.9 (7)	22.2 (6)
If I did not guide or regulate my child’s eating, she/he would eat too many of her/his favourite foods.	20.0 (3)	0 (0)	20.0 (3)	33.3 (5)	26.7 (4)	11.1 (3)	7.4 (2)	25.9 (7)	25.9 (7)	29.6 (8)
**Pressure to eat**										
My child should always eat all of the food on his/her plate.	6.7 (1)	6.7 (1)	20.0 (3)	26.7 (4)	40.0 (6)	3.7 (1)	7.4 (2)	11.1 (3)	37.0 (10)	40.7 (11)
I have to be especially careful to make sure my child eats enough	0 (0)	13.3 (2)	20.0 (3)	40.0 (6)	26.7 (4)	11.1(3)	11.1 (3)	3.7 (1)	25.9 (7)	48.1 (13)
If my child says I’m not hungry, I try to get him/her to eat anyway.	46.2 (6)	7.7 (1)	38.5 (5)	7.7 (1)	0 (0)	66.7 (18)	22.2 (6)	3.7 (1)	3.7 (1)	3.7 (1)
If I did not guide my child’s eating, she/he would eat much less than she/he should.	21.4 (3)	28.6 (4)	14.3 (2)	21.4 (3)	14.3 (2)	44.4 (12)	22.2 (6)	18.5 (5)	7.4 (2)	7.4 (2)
**Concern about child weight**	Extremely unconcerned	Unconcerned	Slightly concerned	Moderately concerned	Extremely concerned	Extremely unconcerned	Unconcerned	Slightly concerned	Moderately concerned	Extremely concerned
How concerned are you about your child eating too much when you are not around her/him?	6.7 (1)	13.3 (2)	26.7 (4)	13.3 (2)	40.0 (6)	18.5 (5)	0 (0)	18.5 (5)	25.9 (7)	37.0 (10)
How concerned are you about your child having to diet to maintain a desirable weight?	20.0 (3)	6.7 (1)	26.7 (4)	6.7 (1)	40.0 (6)	25.9 (7)	0 (0)	18.5 (5)	25.9 (7)	29.6 (8)
How concerned are you about your child becoming over weight?	6.7 (1)	13.3 (2)	0 (0)	20.0 (3)	60.0 (9)	7.4 (2)	0 (0)	11.1 (3)	18.5 (5)	63.0 (17)

**Table 9:** Medians and interquartile ranges (IQR) of CFQ total and three subscale scores.

Subscale Category	Total Sample (n = 82) Median (IQR)	Males (n = 54) Median (IQR)	Females (n = 28) Median (IQR)	*p* Value
Restriction (8 items)	3.3 (1.0)	3.3 (1.2)	3.3 (1.0)	0.90
Pressure to eat (4 items)	3.0 (0.8)	3.1 (1.4)	3.0 (0.6)	0.90
Concern about child weight (3 items)	3.7 (2.3)	4.0 (2.7)	3.7 (2.0)	0.86
Total	3.2 (0.9)	3.2 (1.2)	3.2 (0.7)	0.89

**Table 10:** Spearman’s rank correlation coefficients (r) of physical status, child feeding problems and parent-child feeding relationship variables in the participants (n = 78).

Variable	Sex	Age	Weight	Height	BMI	Total-CFQ	Restriction	Pressure	Concern	Total-STEP	Chewing	Rapid Eating	Food Refusal	Food Selectivity	Vomiting
**Age**	−0.19	--	--	--	--	--	--	--	--	--	--	--	--	--	--
**Weight**	−0.15	0.89 **	--	--	--	--	--	--	--	--	--	--	--	--	--
**Height**	−0.21	0.93 **	0.93 **	--	--	--	--	--	--	--	--	--	--	--	--
**BMI**	0.04	0.65 **	0.83 **	0.63 **	--	--	--	--	--	--	--	--	--	--	--
**Total-CFQ**	0.02	0.06	0.14	0.03	0.31 **	--	--	--	--	--	--	--	--	--	--
**Restriction**	0.02	0.02	0.08	0.01	0.18	0.80 **	--	--	--	--	--	--	--	--	--
**Pressure**	0.01	−0.00	−0.05	−0.10	0.08	0.58 **	0.22	--	--	--	--	--	--	--	--
**Concern**	0.01	0.17	0.31 **	0.18	0.41 **	0.65 **	0.26 *	0.20	--	--	--	--	--	--	--
**Total-STEP**	−0.02	−0.26 *	−0.30 **	−0.30 **	−0.18	0.19	0.20	0.13	−0.04	--	--	--	--	--	--
**Chewing**	−0.03	−0.33 **	−0.42 **	−0.37 **	−0.29 **	0.02	0.02	0.25 *	−0.27 *	0.60 **	--	--	--	--	--
**Rapid eating**	0.08	0.00	0.01	−0.07	0.11	0.32 **	0.41 **	0.06	0.09	0.61 **	0.11	--	--	--	--
**Food refusal**	0.01	−0.17	−0.21	−0.20	−0.17	0.22	0.19	0.10	0.04	0.76 **	0.30 **	0.44 **	--	--	--
**Food selectivity**	−0.08	−0.11	−0.15	−0.11	−0.13	0.11	−0.02	0.11	0.12	0.65 **	0.28 *	0.23 *	0.52 **	--	--
**Vomiting**	−0.11	−0.17	−0.16	−0.18	−0.13	0.00	−0.01	0.14	−0.08	0.48 **	0.22	0.33 **	0.22	0.25 *	--
**Stealing**	0.10	0.05	0.15	0.05	0.21	0.17	0.21	−0.11	0.20	0.34 **	−0.03	0.37 **	0.07	0.02	0.14
* *p* < 0.05		** *p* < 0.001													

Total-CFQ = Total score of Child Feeding questionnaire, Total-STEP = Total score of STEP-CHILD questionnaire.

**Table 11:** Multivariate binary logistic regression for prediction of BMI in the participants.

Variable	B	OR	*p* Value	95% CI for OR	
				Lower	Upper
Concern about child weight	0.34	1.40	0.02	1.06	1.86
Chewing problem	−0.08	0.92	0.62	0.66	1.27
Participant age	−0.03	0.96	0.58	0.84	1.09
Parent-child feeding questionnaire (CFQ) total score	0.00	1.00	0.99	0.92	1.08

OR = Odds Ratio, CI = Confidence Interval.
